# Significant Reduction of the Friction and Wear of PMMA Based Composite by Filling with PTFE

**DOI:** 10.3390/polym10090966

**Published:** 2018-09-01

**Authors:** Dapeng Gu, Longxiao Zhang, Suwen Chen, Kefeng Song, Shouyao Liu

**Affiliations:** 1 School of Mechanical Engineering, Yanshan University, Qinhuangdao 066004, China; jsgudapeng@ysu.edu.cn (D.G.); 13165563169@163.com (L.Z.); songkefeng1994@163.com (K.S.); liushouyaoemail@sina.com (S.L.); 2 Aviation Key Laboratory of Science and Technology on Generic Technology of Self-lubricating Spherical Plain Bearing, Yanshan University, Qinhuangdao 066004, China; 3 Department of Environmental and Chemical Engineering, Yanshan University, Qinhuangdao 066004, China

**Keywords:** PTFE/PMMA composite, friction, wear, powder/liquid

## Abstract

Polytetrafluoroethylene/Poly(methyl methacrylate) (PTFE/PMMA) composite was prepared by mixing PTFE into PMMA matrix which synthesized by the PMMA powder mixture and methyl methacrylate (MMA) liquid mixture. The effects of the filling mass ratio of PTFE and powder/liquid (P/L) ratio on the friction and wear properties of PTFE/PMMA composites against bearing steel were studied by a ball-on-disk tribometer. Fourier transform infrared (FTIR), field emission scanning electron microscopy (FESEM), and energy dispersive X-ray spectroscopy (EDS) were used to characterize the synthesis of PTFE/PMMA composite. The shore hardness and glass transition temperature (*T*_g_) were obtained respectively by shore hardness tester and differential scanning calorimetry (DSC). The results show that the friction coefficient and wear rate of PMMA based composite, comparing with the unfilled PMMA, can be significantly reduced by filling with PTFE. With the increasing of PTFE filling mass ratio, the wear rate of PTFE/PMMA composite increases. The friction coefficient and wear rate of the unfilled PMMA and PTFE/PMMA composite generally decrease with the P/L ratio increasing. The main wear mechanism of the unfilled PMMA is adhesive wear. While the main wear mechanisms of PTFE/PMMA composites are fatigue wear and abrasive wear.

## 1. Introduction

Poly(methyl methacrylate) (PMMA) is a widely used thermoplastic polymer material. It has excellent mechanical properties, high elastic modulus, good dimensional stability, acid-alkali resistance and good biological properties. However, the high friction and wear of pure PMMA restrict its use as a friction material in mechanical parts such as bearings, shaft sleeves, seals, and so on [1,2,3]. Modification by filling organic, inorganic, or metallic micro/nano particles into a polymer matrix producing a new hybrid system is an effective way to improve the mechanical and tribological properties of polymers [4,5,6,7]. As a result, some attempts on filling modification of PMMA based composites have been carried out [8,9,10,11,12,13]. For example, filling nano-calcium oxide (CaO) [8], zirconium oxide (ZrO_2_) [9], silica [10], and nano-calcium carbonate (CaCO_3_) [11] into PMMA can obviously reduce the wear. While filling carbon nanotubes (CNTs) [12], SiO_2_ + Titanium oxide (TiO_2_) [13] can obviously reduce the friction. So the friction coefficient and wear rate of PMMA based composites which filled with the appropriate fillers could be reduced by the physical and chemical synergistic effect of the reinforced fillers and the PMMA matrix. However, the type, content, size, and so on of the reinforced materials have a great influence on the friction, wear, mechanical, and thermodynamic properties of polymer based composites.

As a kind of typical solid lubricant, polytetrafluoroethylene (PTFE) has the advantages on the low friction coefficient, high temperature resistance, and chemical stability. Wang et al. [14] filled PTFE into polyetheretherketone (PEEK) composite and found that the friction coefficient of PTFE/PEEK was lower than the pure PEEK. Zhang et al. [15] found that the friction coefficient and wear rate of poly(phthalazinone ether sulfone ketone) (PPESK) composite filled with PTFE decreased substantially compared with the neat PPESK. For the carbon fiber (CF)/PMMA composites prepared by physical extrusion, filling PTFE can further reduce friction and wear [16]. The addition of PTFE in polymer matrix helped in the formation of a continuous and uniform transfer film and resulted in a reduction in the friction coefficient [17,18].

Moreover, self-curing PMMA has been used extensively as orthopaedic materials [18]. And it is usually used in the form of a two-component self-polymerising system, mainly consisting of a PMMA powder and an MMA liquid that are mixed at room temperature. The powder/liquid (P/L) ratio has obvious effects on the mechanical properties of PMMA based composites [19,20,21]. But few literatures focus on the influence of P/L on the tribological performance of PMMA based composite. Hence, in this paper, the self-curing PMMA composites filled with PTFE were prepared and the dry tribological performances of PTFE/PMMA composites against bearing steel under different P/L ratios were investigated.

## 2. Experiments

### 2.1. Materials

A commercial acrylic product of PMMA powder was supplied by Arkema, Serquigny, France. The methyl methacrylate (MMA) (≥99.5%) monomer liquid, PTFE (5 μm) powder and Catechol (≥99.5%) were all supplied by Aladdin Reagent Co. Ltd., Shanghai, China. The dibenzoyl peroxide (BPO, ≥98%; Containing water 28–32%), *N*,*N*-dimethyl-*p*-toluidine (Dmpt, 98%) were both supplied by Sinopharm Group Chemical Reagent Co. Ltd., Shanghai, China.

### 2.2. Specimen Preparation

The PMMA matrix was synthesized by a two-component polymerization system, consisting of a PMMA powder mixture and an MMA liquid mixture. In the following experiments, three kinds of the P/L ratios (10/10, 11/9 and 12/8, namely 1.0, 1.22 and 1.5) were selected to study the influence of the P/L ratios on the tribological performance of PMMA based composites. The powder mixture was mainly PMMA powder including a small amount (0.8 wt %) of BPO powder. PMMA and BPO powder were kept in a desiccator for 24 h to remove moisture before used. The liquid mixture was mainly MMA with 2 wt % Dmpt and 0.003 wt % Catechol. The mass ratio of filler (PTFE) is defined as, filler/(filler + the powder mixture + the liquid mixture). The filler and the powder mixture with the corresponding mass were mixed and stirred in a mixer for 30 min. After, the powder mixture (PMMA, BPO and PTFE) was gradually mixed and stirred manually into the liquid mixture. Thus the polymerization reaction of PMMA and MMA will be initiated by the action of PBO and Dmpt. After mixing evenly and before curing, the generated PMMA polymer matrix was poured into the cylindrical rubber mold. The inner diameter of the cylindrical rubber mold is 50 mm and the height is 32 mm. Then, the mold was placed in a vacuum box (<10 Pa) in room temperature for 4 h. The samples of PTFE/PMMA composites were demoulded and obtained.

### 2.3. Friction and Wear Test

Dry friction and wear properties of PTFE/PMMA composites were evaluated by a ball-on-disk tribometer (CSM Instruments, Peseux, Switzerland). A steel ball which fixed in a pin-shaped fixture was used to slide against PTFE/PMMA composite. The diameter of the ball (bearing steel, AISI E52100, hardness of HRC 60–65, Ra < 0.02 um) was 6 mm. The diameter of PTFE/PMMA composite was 50 mm and the height was 12 mm. Before tested, the ball was cleaned for 30 min in alcohol using an ultrasonic cleaner. PTFE/PMMA composites were polished (Ra = 0.4–0.6 μm) by a polishing machine and washed with alcohol and deionized water, then ultrasonic cleaned for 30 min in deionized water. The ball was kept stationary and PTFE/PMMA composite was unidirectional rotated under a prescribed set of working conditions at an ambient temperature of 25 °C. The normal load and the sliding speed were fixed to 5 N and 0.2 m/s, respectively. The total sliding distance was 500 m. Each experiment was repeated three times.

At the end of each test, the evolution of the friction coefficient with the sliding distance was measured directly by the CSM tribometer. The wear volume was measured by a conscan confocal optical profilometer (Anton Paar Compact Platform Company, Graz, Austria). The wear performance was expressed by the specific wear rate calculated by the following equation:

*ω* = Δ*V*/(*FL*) (1)

where *ω* is the specific wear rate in mm^3^/(Nm), Δ*V* is the volume loss in mm^3^, *F* is the applied normal load in N, *L* is the total sliding distance in m.

### 2.4. Characterization

The worn surfaces of PTFE/PMMA composites were examined by field emission scanning electron microscopy (FESEM) (Sigma 500, Carl Zeiss, Oberkochen, Germany). In order to improve the conductivity, the surface of the FESEM sample was treated by spraying gold. Energy dispersive X-ray spectroscopy (EDS) and FESEM were used for the determination of the distribution of PTFE in the PMMA matrix. The Fourier transform infrared (FTIR) spectra (600–4000 cm^−1^) of PMMA based composites were recorded with an attenuated total reflection (ATR) unit by a FTIR Spectrometer (Equinox 55, Bruke Company, Karlsruh, Germany) at room temperature.

Differential scanning calorimetry (DSC) (Model 250, TA Instruments, New Castle, DE, American) was performed to determine the *T*_g_ under a nitrogen flow. The test procedure for determining *T*_g_ is listed as follows. Firstly, the samples were heated from room temperature to 200 °C at a heating rate of 20 °C/min and held at this temperature for 3 min to eliminate thermal history. Then, the samples was cooled to 30 °C at a rate of 20 °C/min and held at this temperature for 3 min. The samples were heated again to 200 °C at 20 °C/min, and cooled to room temperature at the same rate. The data obtained from the second scanning were accepted.

## 3. Results and Discussion

**Figure 1:**
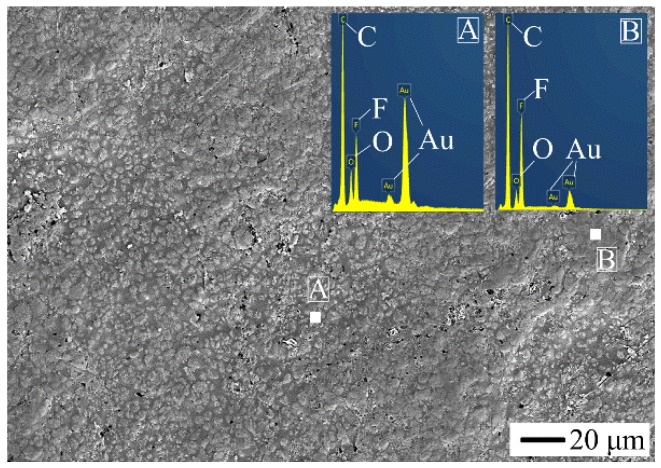
The initial surface morphology of 25 wt % polytetrafluoroethylene (PTFE) filled poly(methyl methacrylate) (PMMA) based composite (powder/liquid (P/L) is 1.0).

**Figure 2:**
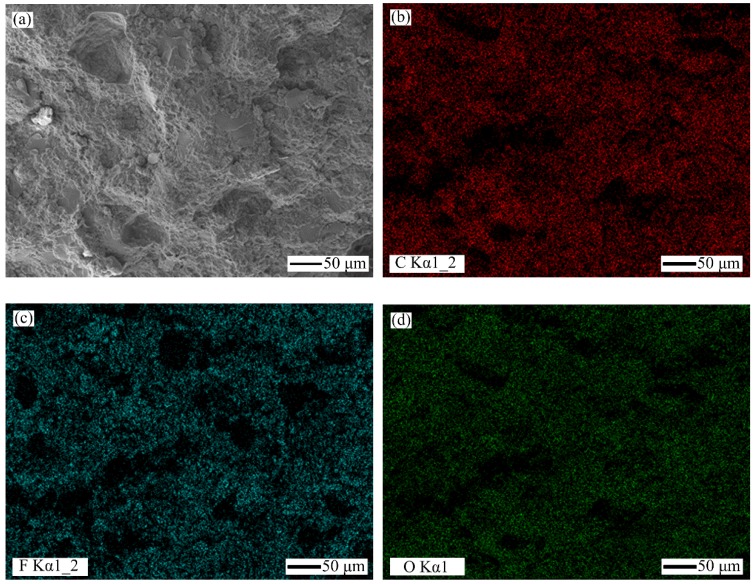
The section’s scanning electron microscopy (SEM) image and energy dispersive X-ray spectroscopy (EDS) mapping of the characteristic elements for 25 wt % PTFE filled PMMA based composite (P/L is 1.22): (**a**) SEM; (**b**) C element; (**c**) F element; and (**d**) O element.

Figure 1 shows the initial surface morphology of 25 wt % PTFE filled PMMA based composite. And Figure 2 shows the scanning electron microscopy (SEM) photo and the distributions of C, F, and O elements of the section of 25 wt % PTFE filled PMMA based composite by EDS. C element is mainly from PMMA matrix and PTFE. O element mainly comes from PMMA matrix. F element originates from PTFE. Au element is originated from the pretreatment of the SEM samples by spraying gold. From the distributions of F and O elements, it can be found that the distribution of PTFE is relatively uniform dispersing in the PMMA matrix.

**Figure 3:**
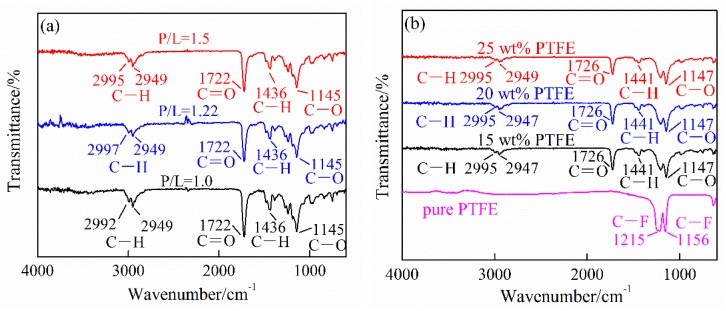
Fourier transform infrared (FTIR) spectra of (**a**) the unfilled PMMA synthesized by the different P/L ratios and (**b**) the PTFE/PMMA composites (P/L = 1.5) and pure PTFE.

Figure 3 shows the FTIR spectra comparison of the unfilled PMMA with different P/L ratios, the PTFE/PMMA composites filled with different PTFE mass ratios (P/L = 1.5) and pure PTFE. The strong vibration bands characteristic to the unfilled PMMA appear at 1722 cm^−1^ (Figure 3a) and to PTFE/PMMA appear at 1726 cm^−1^ (Figure 3b) which both corresponding to the stretching of the C=O group [8,22]. The bands around 1145 cm^−1^ (Figure 3a) to the unfilled PMMA and 1147 cm^−1^ (Figure 3b) to PTFE/PMMA correspond to vibration of the ester group C−O. The bands around 2995 cm^−1^ and 2949 cm^−1^ of both the unfilled PMMA and PTFE/PMMA correspond to the C−H stretching of methyl group (CH_3_). The bands around 1436 cm^−1^ (Figure 3a) to the unfilled PMMA and 1441 cm^−1^ (Figure 3b) to PTFE/PMMA correspond to the C−H shear vibration of CH_3_. It is clearly observed from the spectrum of pure PTFE, the strong absorptions, peaked at 1156 cm^−1^ and 1215 cm^−1^, reflect the C−F symmetrical and asymmetrical of CF_2_ group, respectively [23,24]. Comparing the FTIR spectra of PMMA (P/L = 1.5), pure PTFE and PTFE/PMMA, the absorption spectrum of PTFE/PMMA can be viewed as the superposition of the spectrum of PMMA and that of pure PTFE. And no additional band was observed. So there was no chemical reaction between PTFE and PMMA in the preparation process of PTFE/PMMA composite. PTFE should be dispersed and embedded physically in the generated PMMA matrix and the interaction actions of PTFE and PMMA matrix should mainly be the mechanical anchorage.

**Figure 4:**
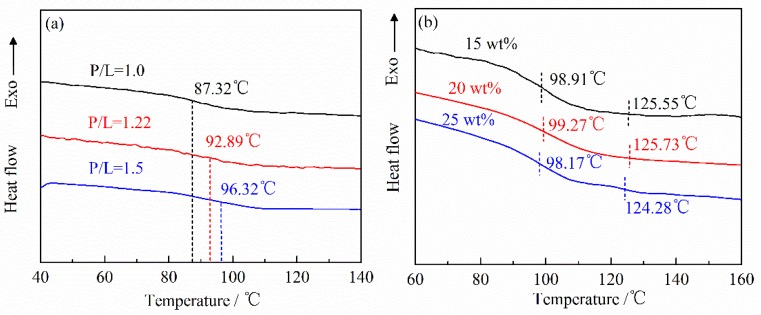
Differential scanning calorimetry (DSC) curves of (**a**) the unfilled PMMA synthesized by the different P/L ratios and (**b**) PTFE/PMMA composites (P/L = 1.5) and pure PTFE.

The glass transition temperatures of the unfilled PMMA, PTFE/PMMA composite, and pure PTFE were obtained by the second scanning after eliminating thermal history. As seen in Figure 4a, the *T*_g_ of the unfilled PMMA increases with the increasing of the P/L ratio. And the highest *T*_g_ is around 96 °C when the P/L ratio is 1.5. The smaller the P/L ratio is, the higher the proportion of the MMA monomer. Massive MMA monomers may not react sufficiently in polymerization process. So the molecular chain which generated with the smaller P/L ratio may be shorter than that of the larger P/L ratio. This may be the reason for the positive correlation between *T*_g_ and the P/L ratio. Figure 4b shows the DSC curves of PTFE/PMMA composites filled with different PTFE mass ratios (P/L = 1.5) and pure PTFE. Due to the complexity of the phase transformation of PTFE, there is still a controversy about the *T*_g_ of PTFE at present, but the second relaxation transformation of PTFE is generally thought to occur between 110–130 °C [25,26,27]. As seen in Figure 4b, it can be seen that PMMA composites filled with PTFE have two glass transitions. The first transformation should be PMMA, and the second may be PTFE. When the filling mass ratio of PTFE is 25 wt %, the two glass transitions are the most obvious, which indicates that PMMA and PTFE are incompatible. Moreover, the *T*_g_ of PTFE/PMMA composites filled with different PTFE mass ratios are approximately the same (all are around 98.8 °C) which are close to that of the unfilled PMMA (96.32 °C).

**Figure 5:**
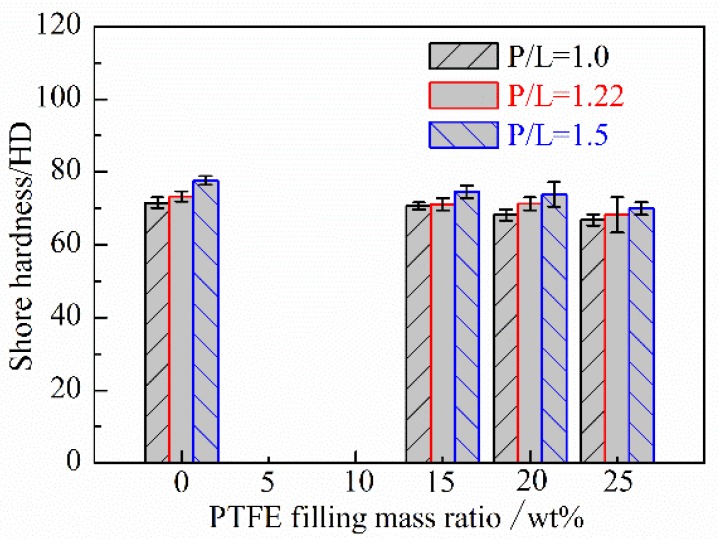
The effect of the filling mass ratio of PTFE and P/L ratio on the shore hardness of PMMA based composites.

The effect of the filling mass ratio of PTFE and P/L ratio on the shore hardness of PMMA based composites are reported in Figure 5. It can be seen that the shore hardness of PMMA based composite decreases generally with the increasing of PTFE filling mass ratio. This is due to the fact that the shore hardness of PTFE is lower than that of PMMA. So the increasing of PTFE filling mass ratio decreases the shore hardness of PTFE/PMMA composite. Moreover, the shore hardness of PMMA based composite increases with the increasing of the P/L ratios under the same PTFE filling mass ratio.

**Figure 6:**
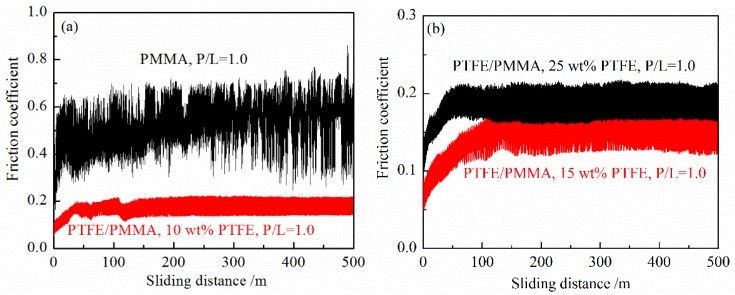
Friction coefficient changes with sliding distance. (**a**) PMMA and 10 wt % PTFE filled PMMA and (**b**) 15 wt % and 25 wt % PTFE filled PMMA.

Figure 6 shows the friction coefficient curves changing with the sliding distance of the unfilled PMMA and PTFE/PMMA composites (P/L = 1.0). On the whole, the fluctuation range of the unfilled PMMA is larger than that of PTFE/PMMA composites. And larger vibrations and noises of the unfilled PMMA were observed during the dry sliding process. However, only slight vibrations were observed of PTFE/PMMA composites. This indicates that the friction process is more stable of PMMA based composites by filling with PTFE.

**Figure 7:**
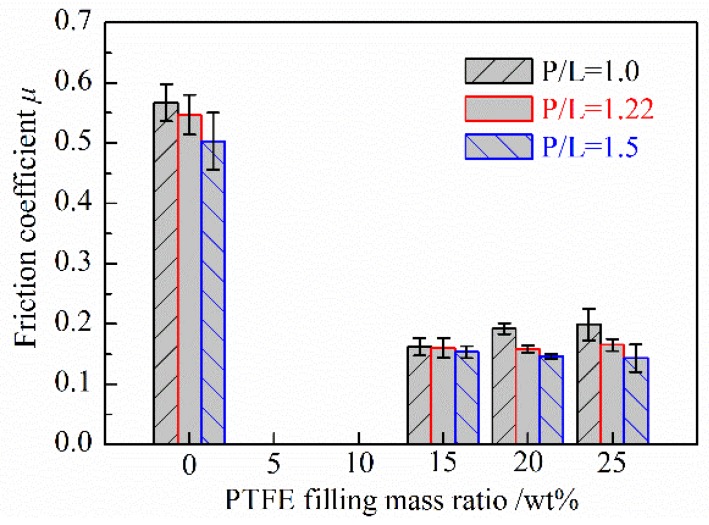
The effect of the filling mass ratio of PTFE and P/L ratio on the friction coefficient of PMMA based composites.

Figure 7 shows the friction coefficients of PMMA based composites with respect to the filling mass ratio of PTFE under different P/L ratios. It can be seen that the friction coefficients of both the unfilled and PTFE filled PMMA based composites decrease with the increasing of the P/L ratios. Compared with the unfilled PMMA, the friction coefficients of PMMA based composites could be significantly reduced nearly two-thirds by filling with PTFE under all three different P/L ratios. Comparing with some previous similar studies, the friction coefficients of PMMA filled with other fillers such as ZrO_2_ [9], Silima [10], CNTs [12], Polyimide + hexagonal boron nitride (PI + hBN) [28], and Zinc oxide (ZnO) [29] are all larger than 0.25, while the friction coefficient of PMMA filled with PTFE is generally lower than 0.2. PTFE is embedded in the PMMA matrix. During the friction process, a PTFE transfer film [30,31,32] with low shear strength is formed in the friction interface playing a role in antifriction and lubricating. So filling PTFE can significantly reduce the friction coefficient of PMMA based composites. In addition, with the increasing of the filling mass ratio of PTFE, the friction coefficients of PTFE/PMMA composites have little change when P/L is 1.22 and 1.5 (in the range of 0.154 ± 0.008). However, the friction coefficient increases instead when P/L is 1.0.

**Figure 8:**
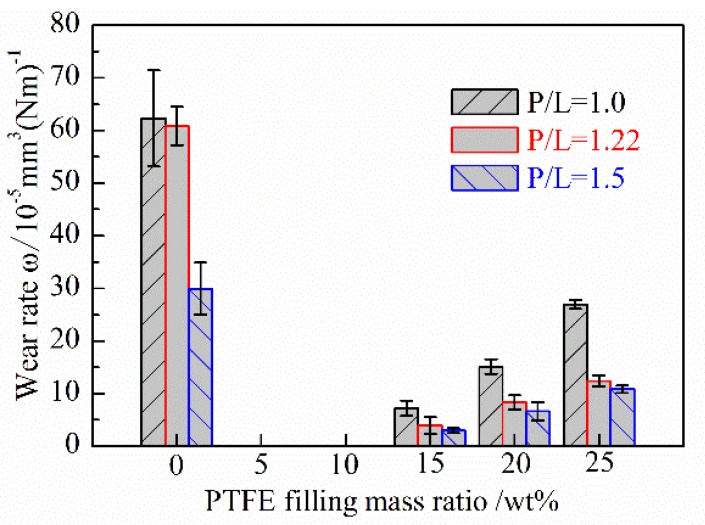
The effect of the filling mass ratio of PTFE and P/L ratio on the wear rate of PMMA based composite.

Figure 8 shows the wear rates of PMMA based composites with respect to the filling mass ratio of PTFE under different P/L ratios. It can be seen that the wear rates of both the unfilled and PTFE filled PMMA based composites decrease with the increasing of the P/L ratios. This may be due to the fact that the shore hardness and *T*_g_ are high when the P/L ratio is high. Thus, the lower wear is obtained for the good heat resistance and anti-adhesion when the P/L ratio is 1.5. The wear rates of PTFE/PMMA composites are far less than that of the unfilled PMMA under all three different P/L ratios. In general, the wear rates of PMMA filled by PTFE are reduced by not less than one half to the unfilled PMMA. Comparing with other similar studies, the antiwear performance of PMMA filled with PTFE is better than PMMA filled with CNTs [12], or PI + hBN [28]. The low friction should be the reason for the low wear of PMMA based composites filled by PTFE. However, as for PTFE/PMMA composites, the wear rates increase with the increasing of the filling mass ratio of PTFE under all three different P/L ratios. That the shore hardness decreases with the increasing of the filling mass ratio of PTFE may be the main cause for this phenomenon.

**Figure 9:**
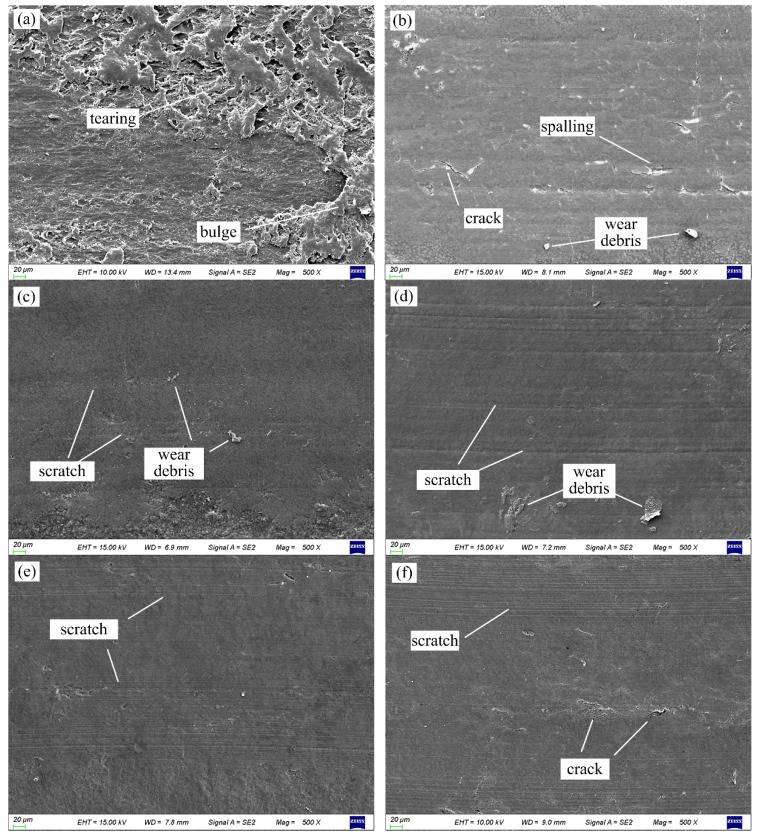
SEM photos of the representative worn surface morphology. (**a**) PMMA, P/L = 1.5, (**b**) 15 wt % PTFE filled PMMA, P/L = 1.5, (**c**) 25 wt % PTFE filled PMMA, P/L = 1.0, (**d**) 15 wt % PTFE filled PMMA, P/L = 1.0, (**e**) 20 wt % PTFE filled PMMA, P/L = 1.22, (**f**) 25 wt % PTFE filled PMMA, P/L = 1.22.

SEM micrographs were performed on the worn surfaces of the unfilled and PTFE filled PMMA based composites for the analysis of the wear mechanisms. As seen in Figure 9a, the worn surface of the unfilled PMMA is uneven, with many tearing flakes and surface bulges. This corresponds to the previous observation of the unstable friction process of the unfilled PMMA. And it also indicates that the adhesive wear mainly occurs on the worn surface of the unfilled PMMA. Figure 9b,c show the worn surface of PTFE filled PMMA based composites with the lowest and highest wear rates, respectively. Figure 9d–f show the representative SEM micrographs with mild wear of PTFE filled PMMA based composites. The worn surfaces of PMMA based composites filled with PTFE are relatively smooth without obvious adhesive wear. This corresponds to the stable friction process of PTFE filled PMMA based composites. But slight scratches on the worn surface can be observed in Figure 9c–f which should be caused by the abrasive wear of the hard particles in the wear debris. In Figure 9b,f, there are some cracks on the worn surface. This indicates that the fatigue wear occurs during the wear process of PTFE filled PMMA based composites.

## 4. Conclusions

(1) The shore hardness of PMMA based composite generally increases with the increasing of the P/L ratio but decreases with the increasing of the filling mass ratio of PTFE. The increasing of the P/L ratio also increases the *T*_g_ of PMMA based composite but the increasing of the filling mass ratio of PTFE has little influence.

(2) Compared with the unfilled PMMA, the friction coefficients and wear rates of PMMA based composites against bearing steel could be significantly reduced by filling with PTFE. With the increasing of the filling mass ratio of PTFE, the wear rates of PTFE/PMMA composites increase and the friction coefficients have little change when P/L is 1.22 and 1.5, but increases instead when P/L is 1.0.

(3) The friction coefficients and wear rates of the unfilled and PTFE/PMMA composites generally decrease with the increasing of the P/L ratios. When the mass ratio of PTFE is 15 wt % and the P/L ratio is 1.5, PTFE/PMMA composites have a lower friction coefficient and the lowest wear rate.

(4) The main wear mechanism of the unfilled PMMA is adhesive wear. While the main wear mechanisms of PTFE/PMMA composites are fatigue wear and abrasive wear.

## References

[1] Cai Z.B., Zhu M.H., Yang S., Xiao X.B., Lin X.Z., Yu H.Y. (2011). In situ observations of the real-time wear of PMMA flat against steel ball under torsional fretting. Wear.

[2] Silvia A.A., Soares B.G., Zaioncz S., Dahmouche K. Poly(methyl methacrylate)-clay nanocomposites prepared by in situ in-tercalative polymerization: The effect of the acrylic acid. Proceedings of the 19 RAU: Annual Meeting of the LNLS Users.

[3] Gross S., Camozzo D., Noto V.D., Armelao L., Tondello E. (2007). PMMA: A key macromolecular component for dielectric low-κ hybrid inorganic-organic polymer films. Eur. Polym. J..

[4] Xu J., Yan H., Gu D. (2014). Friction and wear behavior of polytetrafluoroethene composites filled with Ti_3_SiC_2_. Mater. Des..

[5] Kalin M., Zalaznik M., Novak S. (2015). Wear and friction behavior of poly-ether-ether-ketone (PEEK) filled with graphene, WS_2_ and CNT nanoparticles. Wear.

[6] Pettarin V., Churruca M.J., Felhösb D., Kocsisc J.K., Frontini P.M. (2010). Changes in tribological performance of high molecular weight high density polyethylene induced by the addition of molybdenum disulphide particles. Wear.

[7] Chang L., Zhang Z., Zhang H., Schlarb A.K. (2006). On the sliding wear of nanoparticle filled polyamide 66 composites. Compos. Sci. Technol..

[8] Aguilera-Camacho L.D., Hernández-Navarro C., Moreno K.J., García-Miranda J.S., Arizmendi-Morquecho A. (2015). Improvement effects of CaO nanoparticles on tribological and microhardness properties of PMMA coating. J. Coat. Technol. Res..

[9] Akinci A., Sen S., Sen U. (2014). Friction and wear behavior of zirconium oxide reinforced PMMA composites. Compos. Part B.

[10] Lin L.Y., Kim D.E. (2011). Tribological properties of polymer/silica composite coatings for microsystems applications. Tribol. Int..

[11] Avella M., Errico M.E., Martuscelli E. (2001). Novel PMMA/CaCO_3_ nanocomposites abrasion resistant prepared by an in situ polymerization process. Nano Lett..

[12] Yang Z., Dong B., Huang Y., Liu L., Yan F.Y., Li H.L. (2005). A study on carbon nanotubes reinforced poly(methyl methacrylate) nano-composites. Mater. Lett..

[13] Gu G.T., Zhang Z.J., Dang H.X. (2004). Preparation and characterization of hydrophobic organic–inorganic composite thin films of PMMA/SiO_2_/TiO_2_ with low friction coefficient. Appl. Surf. Sci..

[14] Wang Q.H., Xue Q.J., Liu W.M., Chen J.M. (2000). The friction and wear characteristics of nanometer SiC and polytetrafluoroethylene filled polyetheretherketone. Wear.

[15] Zhang X., Liao G., Jin Q., Feng X., Jian X. (2008). On dry sliding friction and wear behavior of PPESK filled with PTFE and graphite. Tribol. Int..

[16] Wang X.J., Chi Y.L., Cao Y.S. (2014). The effect of PTFE on the mechanical, friction, and wear properties of CF/PMMA composites. J. Thermoplast. Compos. Mater..

[17] Wang Q., Wang H., Fan N., Wang Y., Yan F. (2016). Combined effect of fibers and PTFE nanoparticles on improving the fretting wear resistance of UHMWPE-matrix composites. Polym. Adv. Technol..

[18] He J., Zhang L., Li C., Yan B., Tang R. (2011). The effects of copper and polytetrafluoroethylene (PTFE) on thermal conductivity and tribological behavior of polyoxymethylene (POM) composites. J. Macromol. Sci. Part B Phys..

[19] Silikas N., Kheraif A.A., Watts D.C. (2005). Influence of P/L ratio and peroxide/amine concentrations on shrinkage-strain kinetics during setting of PMMA/MMA biomaterial formulations. Biomaterials.

[20] Samad H.A., Jaafar M. (2009). Effect of polymethyl methacrylate (PMMA) powder to liquid monomer (P/L) ratio and powder molecular weight on the properties of PMMA cement. Polym. Plast. Technol. Eng..

[21] Rodrigues D.C., Gilbert J.L., Bader R.A., Hasenwinkel J.M. (2014). PMMA brush-containing two-solution bone cement: Preparation, characterization, and influence of composition on cement properties. J. Mater. Sci. Mater. Med..

[22] Ahmad S., Ahmad S., Aginhotry S.A. (2007). Synthesis and characterization of in situ prepared poly(methyl methacrylate) nanocomposites. Bull. Mater. Sci..

[23] Padenko E., Van Rooyen L.J., Karger-Kocsis J. (2017). Transfer film formation in PTFE/Oxyfluorinated graphene nanocomposites during dry sliding. Tribol. Lett..

[24] Liang C.Y., Krimm S. (1956). Infrared spectra of high polymers. III. Polytetrafluoroethylene and polychlorotrifluoroethylene. J. Chem. Phys..

[25] Aidani R.E., Dolez P.I., Vu-Khanh T. (2011). Effect of thermal aging on the mechanical and barrier properties of an e-PTFE/Nomex moisture membrane used in firefighters’ protective suits. J. Appl. Polym. Sci..

[26] Calleja G., Jourdan A., Ameduri B., Habas J.P. (2013). Where is the glass transition temperature of poly(tetrafluoroethylene)? A new approach by dynamic rheometry and mechanical tests. Eur. Polym. J..

[27] Gong D., Long J., Fan P., Jiang D., Zhang H., Zhong M. (2015). Thermal stability of micro-nano structures and superhydrophobicityof polytetrafluoroethylene films formed by hot embossing via apicosecond laser ablated template. Appl. Surf. Sci..

[28] Mittal G., Rhee K.Y., Park S.J. (2017). Processing and characterization of PMMA/PI composites reinforced with surface functionalized hexagonal boron nitride. Appl. Surf. Sci..

[29] Chakraborty H., Sinha A., Mukherjee N., Ray D., Chattopadhyay P.P. (2013). A study on nanoindentation and tribological behavior of multifunctional ZnO/PMMA nanocomposite. Mater. Lett..

[30] Zuo Z., Yang Y., Qi X., Su W., Yang X. (2014). Analysis of the chemical composition of the PTFE transfer film produced by sliding against Q235 carbon steel. Wear.

[31] Qiu M., Miao Y., Li Y., Lu J. (2015). Film-forming mechanisms for self-lubricating radial spherical plain bearings with hybrid PTFE/aramid fabric liners modified by ultrasonic. Tribol. Int..

[32] Ren G.N., Zhang Z.Z., Zhu X.T., Men X.H., Liu W.M. (2014). Influence of lubricant filling on the dry sliding wear behaviors of hybrid PTFE/Nomex fabric composite. J. Mater. Sci..

